# Role of TRAFs in Signaling Pathways Controlling T Follicular Helper Cell Differentiation and T Cell-Dependent Antibody Responses

**DOI:** 10.3389/fimmu.2018.02412

**Published:** 2018-10-22

**Authors:** Christophe Pedros, Amnon Altman, Kok-Fai Kong

**Affiliations:** Division of Cell Biology, La Jolla Institute for Allergy and Immunology, La Jolla, CA, United States

**Keywords:** TRAF, follicular helper T cell, antibody response, TCR signaling, costimulation signaling, cytokine signaling, NF-κB

## Abstract

Follicular helper T (T_FH_) cells represent a highly specialized CD4^+^ T cell subpopulation that supports the generation of germinal centers (GC) and provides B cells with critical signals promoting antibody class switching, generation of high affinity antibodies, and memory formation. T_FH_ cells are characterized by the expression of the chemokine receptor CXCR5, the transcription factor Bcl-6, costimulatory molecules ICOS, and PD-1, and the production of cytokine IL-21. The acquisition of a T_FH_ phenotype is a complex and multistep process that involves signals received through engagement of the TCR along with a multitude of costimulatory molecules and cytokines receptors. Members of the Tumor necrosis factor Receptor Associated Factors (TRAF) represent one of the major classes of signaling mediators involved in the differentiation and functions of T_FH_ cells. TRAF molecules are the canonical adaptor molecules that physically interact with members of the Tumor Necrosis Factor Receptor Superfamily (TNFRSF) and actively modulate their downstream signaling cascades through their adaptor function and/or E3 ubiquitin ligase activity. OX-40, GITR, and 4-1BB are the TRAF-dependent TNFRSF members that have been implicated in the differentiation and functions of T_FH_ cells. On the other hand, emerging data demonstrate that TRAF proteins also participate in signaling from the TCR and CD28, which deliver critical signals leading to the differentiation of T_FH_ cells. More intriguingly, we recently showed that the cytoplasmic tail of ICOS contains a conserved TANK-binding kinase 1 (TBK1)-binding motif that is shared with TBK1-binding TRAF proteins. The presence of this TRAF-mimicking signaling module downstream of ICOS is required to mediate the maturation step during T_FH_ differentiation. In addition, JAK-STAT pathways emanating from IL-2, IL-6, IL-21, and IL-27 cytokine receptors affect T_FH_ development, and crosstalk between TRAF-mediated pathways and the JAK-STAT pathways can contribute to generate integrated signals required to drive and sustain T_FH_ differentiation. In this review, we will introduce the molecular interactions and the major signaling pathways controlling the differentiation of T_FH_ cells. In each case, we will highlight the contributions of TRAF proteins to these signaling pathways. Finally, we will discuss the role of individual TRAF proteins in the regulation of T cell-dependent humoral responses.

## Introduction

Production of high-affinity immunoglobulins (Ig) by B cells represents an essential component of protective immunity against pathogens. Antibodies (Abs) function through various mechanisms including specific binding and neutralization of pathogens or toxins, activation of the classical complement pathway, opsonization of pathogens through phagocytosis by innate immune cells, and induction of antibody-dependent cell cytotoxicity (1). The initial activation of naïve B cell leads to the production of secreted IgM and cell surface-bound IgD. After activation, B cells undergo class-switch and acquire the capacity to produce Abs belonging to the IgA, IgE, or IgG sub-classes, depending on environmental cues. These Ig subclasses, which differ in their heavy chains, function through different mechanisms and provide adaptability in response to the diverse forms of foreign antigens. Activated B cells can also undergo somatic hypermutations in the complementarity determining regions of the antigen-binding fragment (Fab), leading to the generation and selection of Ab-forming B cells expressing high-affinity Ig (1). B cells which lose affinity for their target or acquire autoreactivity during this process are eliminated. These B cell maturation events occur in specialized zones of the secondary lymphoid organs, dubbed the germinal centers (GC). GC B cells can differentiate into long-lived plasma cells, providing long lasting memory, and protection. The initial activation of a naïve B cell is T cell-independent, but the maturation events that lead to the generation of high affinity and long lasting protective Ab responses is critically dependent on help signals delivered by a specific CD4^+^ T cell population, known as follicular helper T (T_FH_) cells. T_FH_ cells are characterized by the expression of the transcription factor Bcl6, the chemokine receptor CXCR5, ICOS and PD-1. They provide B cells with essential maturation signals, promote GC formation and reactions, and govern the development of high-affinity Abs (2–4). Expression of the costimulatory molecule CD40L by T_FH_ cells plays a critical role in B cell activation and maturation, and the production of IL-21 and other cytokines by GC T_FH_ cells influence B cell proliferation, survival and isotype switch.

Deficiency of T_FH_ cells, such as in humans suffering from the X-linked lymphoproliferative disease (XLP) or in *Bcl6*^fl/fl^ *Cd4*^Cre^ mice, results in disruption of GC responses, impaired Ab production, and defective memory formation following immunization or infection (5, 6). In humans, several genetic mutations that affect T_FH_ cell differentiation or function have been associated with primary immunodeficiencies characterized by failure to develop protective antibody responses such as the XLP, hyper-IgM syndrome, and common variable immunodeficiency (CVID) [reviewed in (7)]. On the other hand, dysregulated T_FH_ responses, and uncontrolled GC reactions can lead to the production of autoantibodies implicated in the pathogenesis of several autoimmune diseases including systemic lupus erythematosus (SLE), rheumatoid arthritis (RA), and multiple sclerosis (MS) [reviewed in (7)]. Dysregulated T_FH_ responses can also contribute to allergic responses (8), favor the development of B cell malignancies such as follicular lymphomas (9, 10), and even give rise to several subsets of T cell lymphomas such as angioimmunoblastic T-cell lymphoma, follicular T cell lymphoma, and nodal peripheral lymphoma with T_FH_ phenotype (11, 12). Among the mechanisms that dampen GC reactions and Ab responses, follicular regulatory T (T_FR_) cells represent a highly specialized subpopulation of Foxp3^+^ regulatory T cells (Tregs) that co-express Bcl6 and CXCR5. T_FR_ cells have the ability inhibit T_FH_ and B cell responses occurring in the GC [reviewed in (13)]. In *Bcl6*^fl/fl^ *Foxp3*^Cre^ mice, T_FR_ deficiency leads to the development of late onset spontaneous autoimmune diseases and enhanced susceptibility to Ab-mediated autoimmunity (14). The involvement of T_FR_ cells in the pathogenesis of human autoimmune diseases remains speculative, but alteration of the T_FR_:T_FH_ ratio is observed in the blood of patients suffering from several autoimmune diseases [reviewed in (15)].

In light of the key contributions of T_FH_ cells to immune responses, strategies aimed at promoting T_FH_ responses have the potential to improve protective Ab responses against pathogens and vaccines efficacy. On the other hand, inhibiting T_FH_ development or function could be of use for the treatment of immune-mediated diseases or malignancies where increased T_FH_ and GC activity contribute to the disease development or severity such as myasthenia gravis, autoimmune thyroid disease, SLE or RA. Understanding the mechanisms and intracellular signaling pathways that control T_FH_ differentiation and functions is therefore of paramount importance.

In this review, we will first chronicle the spatiotemporal cellular interactions during the multistage T_FH_ differentiation process. Then, we will review the molecular interactions and the intracellular signaling pathways of the T cell receptor (TCR), costimulatory molecules of the immunoglobulin superfamily (IgSF), and tumor necrosis factor receptor superfamily (TNFRSF), and cytokine signaling that play major roles in the differentiation, maintenance, and functions of T_FH_ cells. In each case, we will discuss the known contribution of the tumor necrosis factor receptor associated factors (TRAF) in these signaling pathways. Members of the TRAF family of proteins (TRAF1–6) have been initially identified for their modulation of signaling cascades downstream of TNFRSF members through their adaptor function and/or E3 ubiquitin ligase activity (16). The TRAF-dependent TNFRSF OX-40 (17, 18), GITR (19), and 4-1BB (20, 21) are implicated in the differentiation and functions of T_FH_ cells. TRAF proteins can also participate in signaling from the TCR and the costimulatory receptor CD28 (22–28), which deliver critical signals leading to the differentiation of T_FH_ cells. Engagement of the CD28-related costimulatory receptor ICOS is critical for T_FH_ differentiation (29–31). ICOS plays an important role in T_FH_ differentiation by recruiting phosphatidylinositol 3-kinase (PI3K) (31). Interestingly, ICOS does not recruit TRAFs directly but its cytoplasmic tail contains a binding motif for the TRAF family member-associated NF-κB activator (TANK)-binding kinase 1 (TBK1). This TBK1-binding motif is also present in TRAF2, 3 and 5, the TRAF proteins known to bind TBK1 (32). The presence of this motif in ICOS and the expression of TBK1 are required for the late step of T_FH_ differentiation (32). Furthermore, TRAF proteins can also interfere with the JAK-STAT pathways that are activated downstream of the IL-2, IL-7, IL-6, IL-21, and IL-27 cytokine receptors (33–36) and might therefore affect T_FH_ development by modulating cytokine signaling.

Following discussion of the surface receptors regulating T_FH_ development, we will summarize the TRAF-dependent canonical and non-canonical NF-κB pathways that lead to the differentiation and functions of T-dependent Ab responses. Finally, we will focus on the role of individual TRAF proteins in the regulation of T cell-dependent humoral responses, and discuss their potential contributions at the mechanistic level based on their involvement in the multiple signaling pathways that affect humoral responses.

### Cellular interactions in T_FH_ differentiation

Differentiation of T_FH_ cells is a complex multistep process. It involves sequential interactions between CD4^+^ T cells and professional antigen-presenting cells (APC), namely, dendritic cells (DCs), and B cells. Using traceable immunization and pathogen infection models, the T_FH_ differentiation process can be divided into three spatiotemporal phases: (1) Initiation of T_FH_ differentiation by DC priming of naïve CD4^+^ T cells in the T cell zone of the secondary lymphoid organs; (2) T_FH_ maturation induced by interactions with cognate B cells at the T-B border; and (3) the functional/maintenance phase, within the GC [reviewed in (37, 38)]. The antigen-specific interactions between developing T_FH_ and B cells provide a bidirectional communication that is critical for the maturation of both adaptive immune cells.

During the first few days (days 1–3) following immunization or viral infection, DCs, which are activated at the inflammatory site, enter secondary lymphoid organs and present the engulfed foreign peptides to naïve T cells at the interfollicular and paracortical T cell zones (39). Naïve T cells recognizing the peptide-MHC complex are activated and primed, leading to the induction of the transcription factor Bcl6 (40, 41). Bcl6, the master regulator of T_FH_ cells, is a transcriptional repressor that antagonizes the expression of other lineage-specific transcription factors (42) and microRNAs (43). Bcl6 represses CCR7, the chemokine receptor for the chemokine CCL19 and CCL21 predominantly expressed in the T cell zone, and indirectly promotes the expression of the chemokine receptor CXCR5, the receptor for CXCL13 produced within the B cell zones. As a result of this shift in surface chemokine receptors, these Bcl6^+^CXCR5^+^ pre-T_FH_ cells are no longer retained in the T cell zones, but are attracted along the CXCL13 chemokine gradient toward the T-B border (44). Several costimulatory molecules, such as ICOS, OX40, and CD40L, are also upregulated at the priming stage, regulating the migration, differentiation, and commitment to the T_FH_ cell fate.

During the following few days (day 4–6), the second step of T_FH_ differentiation begins at the T-B border, where pre-T_FH_ cells seek out and interact with cognate B cells. Successful interactions with B cells provide pre-T_FH_ cells with critical signals that ensure the continuation of T_FH_ differentiation programming. During this stage, the expression of Bcl6 and CXCR5 continues to rise, promoting the migration of T_FH_ cells deeper into the B cell follicles, and acquisition of the capacity to help B cells (45). In turn, B cells receive reciprocal signals from differentiating T_FH_ cells, promoting their maturation and entry into the B cell follicles. Only stable T-B conjugates further migrate into the GC (46).

The third phase (day 7 and beyond following immunization or infection) occurs within the GC. Fully differentiated T_FH_ cells localized in the B cell follicles, dubbed GC T_FH_ cells, are characterized by the highest expression of CXCR5 and Bcl6 as well as high expression of PD-1 (44). Through their high expression of CD40L and production of the cytokines IL-4 and IL-21, GC T_FH_ cells control GC B cell proliferation and survival, and drive affinity maturation and the generation of memory B cells. GC T_FH_ can express IL-21 or IL-4 alone or in combination. IL-21-producing T_FH_ cells are efficient in promoting somatic hypermutation, whereas IL-4-producing GC T_FH_ have higher CD40L expression and are able to induce isotype switching and plasma cell differentiation (47). GC T_FH_ cells require continuous antigenic stimulation for their maintenance. In the presence of further antigenic stimulation, long-lived memory T_FH_ cells can persist and rapidly recall the T_FH_ program upon reactivation (48).

The aforementioned cellular interactions between T cells and APCs influence T_FH_ differentiation through a variety of signals delivered through engagement of the TCR, costimulatory molecules, and cytokine receptors. We will first discuss the molecules at play, and then review the implication of TRAF proteins in their signaling pathways.

## Molecular interactions in T_FH_ differentiation and functions

**Figure 1:**
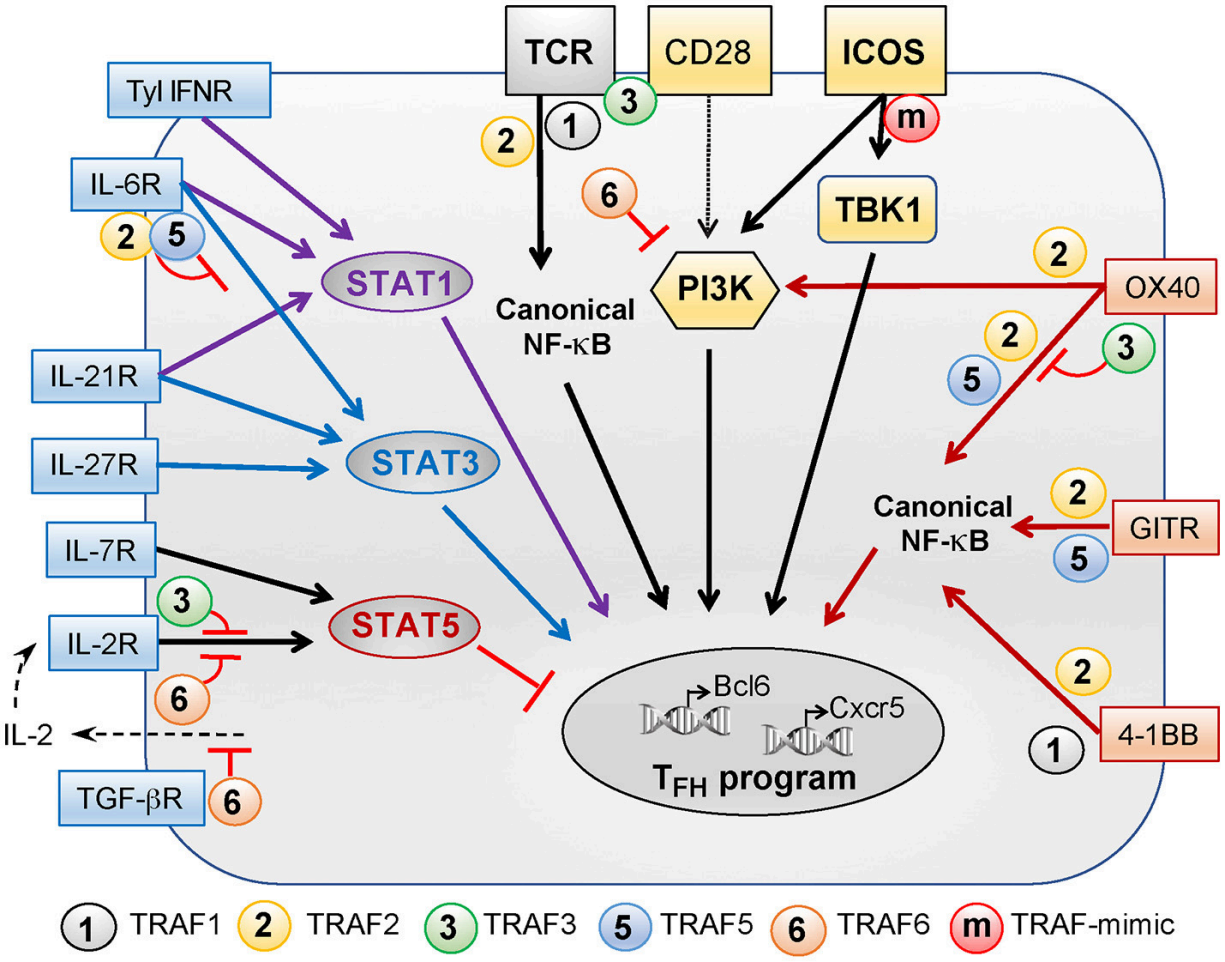
Role of TRAFs in TCR-, costimulatory receptor-, and cytokine receptor-associated signaling pathways controlling T_FH_ differentiation. T_FH_ differentiation depends on a variety of signals received through the TCR, costimulatory receptors of the Ig superfamily (yellow boxes), costimulatory proteins of the TNF receptor superfamily (orange boxes), and cytokine receptors (blue boxes). TRAF1, −2, −3, and −6 regulate TCR signals but TRAF3 activation depends on additional CD28 signaling. TRAF2 contributes to activation of the canonical NF-κB pathway that is critical for T_FH_ differentiation. CD28 and ICOS plays a key role in T_FH_ differentiation and can both activate PI3K. PI3K activation by ICOS is critical for T_FH_ differentiation as compared to CD28-induced PI3K signals. Recently, we revealed that the TANK-binding kinase TBK1 functions downstream of ICOS to promote T_FH_ differentiation. TRAFs are not recruited to ICOS but the presence of a TRAF-like motif in the intracellular tail of ICOS bypasses the need for TRAFs to recruit TBK1. The TNFR family members OX40, GITR, and 4-1BB signal through TRAFs and also contribute to T_FH_ differentiation. TRAF2 is involved in recruitment and activation of PI3K by OX40. TRAF2 and−5 promote NF-κB pathway activation downstream OX40 and GITR while TRAF1 and−2 associate with 4-1BB to promote this pathway. TRAF3 plays a regulatory role in OX40 signaling. Signals through type 1 IFN, IL-6, and IL-21 receptors converge through STAT1 activation, positively regulating T_FH_ differentiation. Signaling through IL-6, IL-21, and IL-27 receptors activates STAT3 to promote T_FH_ differentiation. TRAF2 and−5 can both inhibit IL-6 mediated activation of STAT3. STAT5, a negative regulator of T_FH_ differentiation, can be activated by signals through the IL-2 and IL-7 receptors. TRAF3 and−6 both negatively regulate IL-2R-induced signaling. Finally, TRAF6 is involved in the suppression of IL-2 production in the presence of TGF-β and, thus, could indirectly promote early T_FH_ differentiation by limiting signals received through the IL-2R.

Upon sequential interactions with DCs and B cells, the T_FH_ differentiation program is initiated and maintained through integration of multiple signals received from the TCR, costimulatory and coinhibitory receptors, and cytokine receptors. In this part, we will review the role of these signals in T_FH_ differentiation and the contribution of TRAFs in the signaling pathways that they trigger (summarized in Figure 1).

### TCR signaling

Engagement of the TCR is the initial and central event that triggers naïve T cell activation and differentiation. Together with other factors, including engagement of costimulatory or inhibitory receptors and cytokine signaling, the strength and duration of TCR signals impact the outcome of T cell activation and differentiation.

Using TCR-transgenic T cells with varying binding affinities to a pigeon cytochrome C peptide, it was revealed that T cells with a high-affinity TCR preferentially develop into CXCR5^+^ T_FH_ (49). Concomitantly, a knock-in mouse strain expressing a mutated, non-signaling CD3ζ chain showed a selective defect in the generation of T_FH_ cells (50). However, a high-affinity TCR does not appear to be an absolute prerequisite for T_FH_ differentiation as T_FH_ cells can also be generated after priming with intermediate and low affinity antigens (51). In the latter cases, B cells appear to play a key role in driving the differentiation of T_FH_ cells with low TCR affinity (51). Additionally, experiments with different doses of antigen reveal that, for a given TCR affinity, increasing the amount of antigen available (45, 52) or a second peptide immunization that prolong antigen presentation (53) favors T_FH_ differentiation. In contrast, another group demonstrated that the differentiation of T_FH_ cells is reduced upon immunization with high doses of strong agonist peptide, as compared to lower doses (54). Differences in the inflammatory environment generated by the different antigen delivery systems might therefore influence the strength of TCR signals in favoring or antagonizing T_FH_ differentiation. Taken together, our current understanding is that strong and sustained TCR–DC interactions promote T_FH_ differentiation. Indeed, intravital imaging analysis reveals that sustained T-DC interactions promote T_FH_ differentiation (52, 55).

TRAF1, 2, 3, or 6 can positively or negatively modulate signaling downstream of the TCR-CD3 complex (Figure 1). For example, *Traf1*^−/−^ CD8^+^ T cells exhibit increased levels of active p52 after anti-CD3 stimulation, indicating that TRAF1 restrains the activation of the non-canonical NF-κB pathway in the absence of costimulation (23). As a result, *Traf1*^−/−^ T cells hyperproliferate in response to stimulation with anti-CD3 Ab (22, 23). In contrast, *Traf2*^−/−^ CD4^+^ T cells show reduced proliferation and activation after *in vitro* anti-CD3 stimulation (24). TRAF2 plays a positive role in the regulation of NF-κB signaling as *Traf2*^−/−^*Tnf* ^−/−^ T cells display a constitutively active non-canonical NF-κB pathway (56). In the absence of TRAF3, T cells exhibit reduced proliferation and cytokine production following costimulation with anti-CD3/CD28 Abs, reflecting an impaired activation of TCR signaling molecules Zap70, LAT, Erk, and PLCγ1 (25). Furthermore, TRAF3 has been shown to sequester the membrane localization of the kinase Csk and the phosphatase PTPN22, two known inhibitors of the TCR signaling, thereby reducing the threshold of T cell activation (26). On the other hand, *Traf6*^−/−^ T cells hyperproliferate *in vitro* in response to stimulation with anti-CD3 Ab alone, bypassing the requirement for costimulation. Interestingly, the NF-κB pathway is independent of TRAF6. Instead, *Traf6*^−/−^ T cells exhibit constitutive activation of phosphatidyl-inositol 3-kinase (PI3K), demonstrating that TRAF6 negatively regulates PI3K signaling following TCR engagement (27). In addition, TRAF6 can also be recruited to the T cell immunological synapse through the adaptor molecule LAT, promoting its ubiquitination and phosphorylation and positively regulating the activation of the calcium-sensing transcription factor, nuclear factor of activated T cells (NFAT) (28). Hence, it is becoming increasingly apparent that TRAF1, 2, 3, and 6 can influence the quality and intensity of TCR signaling through various mechanisms. However, it remains to be determined whether this TRAF-dependent modulation of TCR signaling is necessary and/or sufficient to significantly impact the differentiation of T_FH_ cells.

### Costimulatory signaling

#### CD28 signaling

Activated DCs present pathogen-derived peptide antigens associated with MHC class II molecules and upregulate the costimulatory ligands CD80 and 86, which interact with the costimulatory receptor CD28 on T cells. Interestingly, there is a selective preference for CD86 over CD80 to induce the formation of T_FH_ cells (57, 58). This reflects the fact that CD86 is a higher affinity ligand of CD28 (59). As a result, the CD86-CD28 interaction is less likely to be attenuated by the competing CD86-CTLA-4 interaction, and, therefore, could deliver a more sustained stimulatory signal than CD80.

Signals elicited through CD28 are essential for the activation of naïve T cells and their development into all effector T cell subsets. The differentiation of T_FH_ cells is no exception to this rule. The importance of CD28 for T-dependent Ab responses has been demonstrated using two different genetic models. First, *Cd28*^−/−^ mice are deficient in GC formation and exhibit a delay in serum IgG titers following immunization with the hapten nitrophenol (NP) conjugated to chicken γ-globulin (NP-CGG) (60). Lack of CD28 costimulation in *Cd28*^−/−^ T cells intrinsically inhibits the upregulation of the T_FH_ master transcription factor Bcl-6 and, thus, all subsequent T_FH_ differentiation steps are abrogated (61). Second, using a transgenic mouse strain ectopically expressing the soluble CD28 competitor, CTLA4–IgG fusion protein that blocks the interaction between CD28 and CD80/86, the T cell-dependent GC responses and antigen-specific T_FH_ cells are dramatically attenuated (62, 63). However, this defect can be compensated by the coinjection of the NP-CGG antigen and an agonistic anti-CD28 Ab (63), because the latter bypasses the inhibitory effect of CTLA4-Ig. On the contrary, GC reactions in CTLA4-Ig mice are not restored when the agonistic anti-CD28 Ab is administered 10 days after immunization (64). Similarly, blocking CD28 by injection of CTLA4-Ig in wt mice 6–7 days post-immunization does not negatively impact T_FH_ differentiation (61). Altogether, these results suggest that CD28 plays a key role during early T cell priming but not during the later phase of T_FH_ maturation or maintenance in the GC. Consistent with this notion, the absence of CD80 specifically on DCs abolishes T_FH_ differentiation whereas the absence of CD80 expression on B cells does not (65).

The signaling events that mediate CD28 function have been extensively studied, and signaling molecules that bind to specific motifs within the cytoplasmic tail of CD28 have been identified. The proximal tyrosine motif (YMNM) binds and activates the p85α subunit of PI3K as well as other adaptor proteins, including Grb2 and GADS. The distal proline-rich motif (PYAP) binds and activates Src family kinases and, indirectly, protein kinase C-θ (PKCθ) (66, 67). Using knock-in mouse strains expressing CD28 with mutations in either the proximal tyrosine motif or the distal proline-rich motif, it was demonstrated that the formation of GC and isotype switching are dependent on the PYAP motif, whereas the PI3K-binding YMNM sequence is dispensable (68). These results imply that CD28-mediated Lck and PKCθ signaling are critical for T_FH_ differentiation. However, PI3K signaling mediated by CD28 is less important than PI3K signaling emanating from ICOS (see below).

#### ICOS signaling

In humans, ICOS deficiency results in severe impairment of germinal center formation and inability to mount antibody responses against infection or vaccination (69, 70). Since its initial characterization (71), it has been established that ICOS is a major driver of T-dependent Ab responses and GC reactions. *Icos*^−/−^ mice have defective GCs, impaired humoral response to antigens, and lack immunological memory (72–74). Similarly, ICOS ligand (ICOSL) deficiency or blockade of ICOS-ICOSL interaction using an anti-ICOSL Ab strongly reduces T_FH_ development (29, 30). However, the temporal requirement for ICOS signals during the complex T_FH_ differentiation process appears to vary depending on the experimental model. In an acute infection model, ICOS is required for the early CXCR5^+^Bcl6^+^ T_FH_ differentiation of antigen-specific T cells as early as 3 days following infection with lymphocytic choriomeningitis virus (LCMV) (32, 75). Consistent with ICOS signaling during the early DC-T cell engagement favoring T_FH_ differentiation through Bcl6 induction, ICOSL expression on CD8α^−^ DCs favors the initiation of CXCR5^+^Bcl6^+^ T_FH_ differentiation (76). In stark contrast, the early expression of Bcl6 by ovalbumin-specific OT-II CD4^+^ T cells is not affected by ICOS deficiency 3 days following NP-OVA immunization (61). Similarly, *Icos*^−/−^ mice show intact T_FH_ differentiation for as long as 6 days following infection with the non-lethal strain of malaria, *Plasmodium chabaudi* (77), indicating that early T_FH_ differentiation can occur in an ICOS-independent manner in some models.

In addition to the priming stage, the ICOS-ICOSL interaction between T_FH_ and B cells is also required for the maturation of developing T_FH_ cells. Administration of an anti-ICOSL blocking Ab drastically curtails the T_FH_ cell population in various infection models (61, 75, 77). Similarly, the expression of ICOSL by B cells is required for the generation of TFH cells (78). Additionally, ICOS is required for close contacts between T and B cells in the GC, promoting the expression of CD40L at the T cell surface and delivery of contact-dependent help to B cells (79).

##### ICOS-mediated activation of PI3K

PI3K signaling has been implicated as an important mediator downstream of several T cell molecules (TCR, CD28, CTLA-4, and ICOS). PI3K is a heterodimer consisting of a p110 catalytic subunit (of either the α, β, γ, or δ isoform) and a regulatory subunit, which can be p85α, p55α, p50α, p85β, or p55γ. The relevance of the ICOS-mediated PI3K signaling in the differentiation of T_FH_ cells has been elegantly demonstrated using a knock-in mouse strain expressing an ICOS mutant incapable of binding PI3K (ICOS-YF). Similar to *Icos*^−/−^ mice, ICOS-YF knock-in mice fail to generate T_FH_ cells and GC reactions (31). The phenotype of ICOS-YF mice is in stark contrast to the CD28-YF mice, which are capable of mounting T cell-dependent Ab responses (68). This is consistent with the fact that ICOS delivers a more potent PI3K signaling than CD28 in T cells (80).

Although the PI3K-binding motifs of CD28 and ICOS differ by a single amino acid, i.e., YMNM in CD28 and YMFM in ICOS, the resulting difference in hydrophobicity property of these motifs confers a significant alteration in T cell signaling (81). ICOS triggering not only promotes the physical interaction between ICOS and the PI3K regulatory subunits p85α and p50α in activated T cells, but also promotes their recruitment to CD28 “in trans,” in the absence of CD28 ligation (80). Because p50α is the most potent isoform in regulating the kinase activity of PI3K (82, 83), ICOS ligation induces a higher PI3K activity as compared with CD28 ligation and delivers a more potent costimulatory signal favorable for the differentiation of T_FH_ cells.

To understand the role of PI3K in the generation of humoral responses, several complementary approaches have been used. First, Ab responses, including isotype switching, GC formation, and GC B cells, are severely impaired in *p110*δ^−/−^ mice following hapten-induced T-dependent and T-independent challenges (84). Second, using a mouse strain expressing a catalytically inactive form of p110δ (p110δ^D910A^), but intact (active) p110α, and p110β isoforms, the abrogation of p110δ lipid kinase activity alone was sufficient to result in a near complete absence of GC and a profound reduction of serum IgG titers following immunization with T-dependent or T-independent antigens (85). However, these initial observations are confounded by the combined functional defects in T and B cell compartments. Third, a T cell-specific deletion of the p110δ catalytic subunit in *p110*δ^fl/fl^ *Cd4*^Cre^ mice results in a nearly absence of CXCR5^+^PD-1^+^ GC T_FH_ cells, and a significant reduction of GC B cells, GC reactions, and Ab affinity maturation following immunization with NP conjugated to keyhole limpet hemocyanin (KLH) (86). These findings reveal the non-redundant and T cell-intrinsic role of p110δ in T_FH_ cell development. Fourth, *p110*δ^fl/fl^ *Ox40*^Cre^ mice show similar defects in humoral responses following immunization (86). Since OX40 is expressed following TCR- and CD28-mediated T cell activation, ablation of p110δ at this later time point indicates that this catalytic subunit is crucial for T-dependent Ab responses after the initial activation of naïve T cells (86). Fifth, the magnitude and output from GC reactions are unperturbed in immunized *p110*δ^fl/fl^ *Cd19*^Cre^ mice (86), implying that p110δ is dispensable in B cells, and/or that other PI3K catalytic subunits may contribute in a redundant manner to the GC reactions. Sixth, combined deletion of genes that encode four PI3K regulatory isoforms normally expressed in T cells (p85α, p55α, p50α, p85β) results in a drastic deficiency in T cell help to B cells *in vivo*. These mice display a significant reduction in GC numbers and size, as well as the production of class-switched Abs following immunization (87). Taken together, these data indicate that the ICOS-mediated PI3K pathway is crucial for T-dependent Ab responses.

##### Importance of PI3K-independent ICOS signaling

The PI3K-binding YMFM motif is a crucial feature of ICOS signaling in mediating the differentiation and functions of T_FH_ cells. However, the knock-in ICOS-YF mouse strain, in which the association between ICOS and PI3K is selectively lost, is not a true phenocopy of *Icos*^−/−^ mice (31). For example, in a model of respiratory infection with *Chlamydia muridarum*, ICOS-YF mice develop a much milder disease as compared to *Icos*^−/−^ mice, albeit they are still not fully protected (88). Th17 responses negatively correlate with disease severity and are strongly reduced in *Icos*^−/−^ mice but partially retained in ICOS-YF mice. Similarly, the severity of graft-vs.-host disease in ICOS-YF mice is intermediate between wild-type (wt) and *Icos*^−/−^ mice, in a model of MHC-mismatched bone marrow transplantation (89). Interestingly, in this model, CD8^+^ T cells from ICOS-YF mice induce a disease indistinguishable from that induced by wt CD8^+^ T cells, whereas ICOS-YF and *Icos*^−/−^ CD4^+^ T cells behave similarly. *In vitro*, ligation of ICOS induces T cell activation, calcium flux and proliferation of CD8^±^ T cells in a PI3K-independent manner (89). Similarly, the PI3K-independent role of ICOS in activating calcium flux was demonstrated in CD4^±^ T cells (31). Altogether, these data strongly evince the presence of important PI3K-independent pathway(s) downstream of ICOS.

##### TRAF-mimicking ICOS signaling

The aforementioned studies also pose a conundrum because other than its PI3K-binding motif, the cytoplasmic tail of ICOS lacks canonical motifs for protein-protein interactions. To resolve this issue, we looked for potential evolutionarily conserved sequence(s) in the cytoplasmic tail of ICOS (32). Remarkably, in addition to the YMFM motif, we found two additional highly conserved motifs in the intracellular domain of ICOS. They are the IProx motif (SSSVHDPNGE) and a more distal motif (AVNTAKK). Using an unbiased proteomics approach, TANK-binding kinase 1 (TBK1), a non-canonical member of the inhibitor of transcription factor NF-κB kinase (IKK) family, was unexpectedly found to interact with the serine-rich IProx motif. Mutation of this specific motif abrogated TBK1 binding to ICOS, but did not affect ICOS ability to recruit PI3K (32).

Similar to the mutation of the PI3K-binding motif (ICOS-YF), *Icos*^−/−^ CD4^+^ T cells reconstituted with a mutated IProx motif (mIProx) displayed impaired CXCR5^+^PD-1^+^ GC T_FH_ differentiation, GC formation, and IgG responses. Moreover, TBK1 knockdown in T cells resulted in defective humoral responses in response to acute LCMV infection (32). Although *Icos*^−/−^ CD4^+^ T cells reconstituted with an ICOS-YF mutant fail to generate nascent CXCR5^+^ Bcl6^+^ T_FH_ cells, this initial step of T_FH_ development was not compromised in T cells expressing mIProx (32). Consistently, TBK1 was also dispensable for the development of nascent T_FH_ cells, indicating that signals mediated by TBK1 binding to the ICOS IProx motif 'license' nascent T_FH_ cells to enter the GC phase of T_FH_ cell development. In agreement with our findings, it has recently been demonstrated that therapeutic inhibition of TBK1 reduced the number of GC T_FH_ and their expression of *Bcl6*, caused a reduction in GC size, diminished the anti-collagen Ab levels and alleviated the progression of established collagen induced arthritis (90).

In contrast to the interaction between ICOS and PI3K, which can be induced by anti-CD3 or anti-ICOS stimulation alone, TBK1 coimmunoprecipitates with ICOS only when T cells are stimulated with a combination of anti-CD3 plus anti-ICOS Abs (32). These combined stimuli are physiologically provided by the strong cognate interaction that occurs *in vivo* between T cells and APCs. Hence, the requirement for activation of ICOS-TBK1 signaling is more stringent than that for the ICOS-PI3K pathway.

We further found that despite the known ability of TRAF2, 3, and 5 to physically interact with TBK1 (91–93), these TRAFs proteins were not corecruited with TBK1 to ICOS upon stimulation (32). Unexpectedly, the serine-rich IProx motif in ICOS turned out to be highly homologous with a region of TRAF2 and TRAF3 known as the “serine tongs,” which consists of the sequence SSSxxxPxGD/E (where 'S' is serine, “x” is any amino acid, “P” is proline, “G” is glycine and “D/E” indicates aspartic acid or glutamic acid). Substitution of this region in TRAF2 and TRAF3 with a string of alanines abolished their ability to bind TBK1. Thus, this sequence, which is also present in a similar form in the cytoplasmic region of ICOS, represents a previously unknown consensus TBK1-binding motif. The presence of this motif in ICOS therefore allows it to directly recruit TBK1, obviating the need for TRAF proteins as intermediary partners for TBK1 activation (Figure 1).

##### ICOS-dependent calcium signaling

The ability of ICOS to potentiate TCR-induced calcium flux is conserved in *Icos*^−/−^ CD4^±^ T cells expressing an ICOS mutant where most of the cytoplasmic tail is truncated, including the PI3K and TBK1 binding motifs (94), demonstrating that the ICOS-triggering calcium flux is independent of PI3K and TBK1. Interestingly, a short membrane anchoring sequence consisting of the sequence KKKY (where “K” is lysine and “Y” is tryptophan) is present in this mutant. Mutation of the KKKY motif in full-length ICOS dampens the calcium response in T cells, showing that this motif is both necessary and sufficient for calcium flux (94). This motif in ICOS is likely to positively regulate T_FH_ responses since ICOS engagement and calcium flux promote CD40L surface expression (79), a critical requirement for T_FH_ to provide B cells with contact dependent help signals.

#### OX40 signaling

CD28 costimulation induces the expression of OX40, a TNFRSF member, on T cells (63). OX40 stimulation is involved in upregulation of *Cxcr5* mRNAs (95), and higher expression of OX40 has been reported on T_FH_ cells (49). However, the degree to which OX40 influences the development of T_FH_ cells and Ab responses is highly context-dependent. *Ox40*^−/−^ mice are able to mount effective humoral responses against acute LCMV, vesicular stomatitis virus (VSV), and influenza A virus infections, suggesting a non-essential role for OX40 in T_FH_ development (96). Similarly, the absence of OX40 does not affect the expression of CXCR5 on antigen-specific CD4^+^ T cells and the development of IgG1 responses after infection with the rodent roundworm *Heligmosomoides polygyrus* (97). In stark contrast, OX40 is required to mount an efficient T_FH_ and humoral response against chronic infection with the Clone 13 strain of LCMV. *Ox40*^−/−^ mice are not able to control viral replication (17). In a *Vaccinia* virus infection model, *Ox40*^−/−^ mice also exhibit a dramatically reduced **T**_FH_ differentiation and Ab response. Blocking experiments using an anti-OX40L Ab showed that the OX40-OX40L interaction is required for both T_FH_ generation and maintenance in this model (18). Variations in the expression of OX40 by CXCR5^+^ CD4 T cells in different mouse strains might account for the differential impact of OX40L Ab treatment on T_FH_ generation and GC responses (29).

OX40 signaling can induce the expression of multiple T_FH_ molecules, including CXCR5 and IL-21, by human T cells, and likely contributes to the pathogenic role of T_FH_ cells in SLE (98). Mechanistically, TRAF2, 3, and 5 are recruited to the cytoplasmic tail of OX40 [Figure 1; (99, 100)]. However, TRAF2 plays a more important role in OX40 signaling by promoting the recruitment of PI3K, AKT, PKCθ, and IKKα, β, and γ, which trigger the mTOR and the canonical NF-κB pathways (99–101).

#### GITR signaling

GITR expression is induced late during the maturation phase of GC T_FH_ cells (102). The number of T_FH_ cells is not affected in *Gitr*^−/−^ mice during the first week of chronic infection with LCMV Clone 13. This is consistent with findings that the initial production of LCMV-specific IgG is not affected. However, Ab titers do not increase in *Gitr*^−/−^ mice beyond the first week of infection. This defect is associated with an increase in the proportion of splenic Foxp3^+^ CXCR5^+^PD-1^+^ T_FR_ cells, and a reduction in Foxp3^−^ CXCR5^+^PD-1^+^ T_FH_ cell numbers, suggesting that GITR plays a role in regulating the ratio between T_FR_ and T_FH_ cells. This GITR-mediated function is T-cell intrinsic because in mixed bone marrow chimera experiments, the T_FH_ cell population is diminished in *Gitr*^−/−^ CD4^+^ T cells, as compared to the wt CD4^+^ T_FH_ cells following chronic LCMV infection (102). Consistent with a role for GITR signals in promoting humoral responses, administration of a recombinant GITR ligand protein enhanced the frequency of CXCR5^+^ICOS^+^ T_FH_ cells and the expression of Bcl6 and IL-21 in a model of collagen-induced arthritis (19). Conversely, blocking GITR signals using a GITR-Fc fusion protein reduced the frequency of T_FH_ cells, IgG production, and disease severity (19).

As a member of the TNFRSF, several TRAF molecules interact with the cytoplasmic tail of GITR. In CD8^+^ T cells, TRAF2 and 5 are involved in activation of the canonical NF-κB pathway triggered by GITR stimulation [Figure 1; (103)]. Additionally, the GITR-TRAF5 axis is known to activate the MAP kinase signaling pathways because *Traf5*^−/−^ CD4^+^ T cells are defective in the activation of p38 and ERK kinases (104). On the other hand, TRAF3 has been demonstrated to inhibit the activation of the non-canonical NF-κB pathway triggered by GITR engagement (105). However, it is unclear which of these TRAF molecule(s) in the GITR signaling pathway plays a more prominent role in the maintenance of T_FH_ cells.

#### CD40L signaling

CD40L is rapidly upregulated upon TCR and costimulatory receptor ligation, and/or cytokine signaling (106). CD40L expressed on T cells is the ligand for the TNFRSF member CD40 expressed on B cells. CD40-CD40L signaling is essential for the development of T cell-dependent humoral responses. *Cd40*^−/−^ or *Cd40l*^−/−^ mice are severely defective in their ability to generate GC or develop IgG responses (107, 108). Similarly, individuals deficient for CD40 or CD40L suffer from hyper-IgM syndrome characterized by elevated IgM level, disrupted GC formation and reduced IgG, IgA and IgE levels (7). The requirement for CD40L signals received by T cells appears to be dispensable for early T_FH_ differentiation during the DC priming phase (75), consistent with the fact that CD40L is highly expressed after the priming phase. Similarly, CD40 expression by DC is dispensable for an efficient T_FH_ and IgG response, whereas CD40 expression on B cells is absolutely required for the generation of GC and T_FH_ development (65). Interestingly, the intrinsic role of CD40L signaling in T cells does not appear to be critical for T_FH_ differentiation as wt and *Cd40l*^−/−^ antigen-specific T cells expand and differentiate into T_FH_ cells to a comparable extent in a cotransfer experiment (65).

Very little is known about CD40L signaling in T cells. However, engagement of B cell-expressed CD40 by CD40L directly or indirectly recruits TRAF1, −2, −3, −5, and −6 to its cytoplasmic domain [reviewed in (109)]. The persistent TRAF-dependent CD40 signaling in B cells, delivered by CD40L expressed by T cells is considered to be one of the most potent signals in mediating different aspects of B cell biology, including differentiation, survival, proliferation, expression of costimulatory molecules, and cytokines, maturation of GC B cells, isotype switching, somatic hypermutation, and formation of long-lived plasma cells and memory B cells.

#### 4-1BB signaling

4-1BB (CD137 or TNFRSF9) is highly expressed on human T_FH_ cells (110). However, *4-1bb*^−/−^ mice show no impairment of IgG production following VSV infection (111). Similarly, the absence of 4-1BB ligand in *4-1bbl*^−/−^ mice does not affect the T cell-dependent Ab responses (112). These data imply that 4-1BB and its ligand might be dispensable for the generation of T cell-dependent humoral responses in rodents. However, *in vivo* treatment with an agonistic anti-4-1BB Ab inhibits T cell-dependent Ab responses in various mouse models (20, 21, 113). *In vivo* administration of an agonistic anti-4-1BB Ab at the time of priming strongly reduces the development of Ab responses to T cell-dependent antigens (20). Additionally, treatment with an agonistic anti-4-1BB Ab suppresses the ongoing CD4^+^ T cell-dependent autoantibody production in the NZB × NZW mouse model of SLE (21). Therefore, excessive 4-1BB signals during both the initiation of Ab responses and their maintenance could negatively modulate T_FH_ differentiation and/or functions. The exact mechanisms by which these agonistic Abs influence T cell-dependent humoral responses warrants careful interpretation because the expression of 4-1BB is not restricted to T cells. For instance, one study suggested that anti-4-1BB Ab treatment blocks GC formation by downregulating the follicular dendritic cell (FDC) network (114), a specialized subset of follicle-residing cells that support the GC reaction.

Cytoplasmic TRAF1 and TRAF2 are recruited to 4-1BB upon stimulation [Figure 1; (100, 115)]. TRAF1 is required for the activation of the classical NF-κB pathway following 4-1BB engagement (23). Following stimulation with an agonistic anti-4-1BB Ab, 4-1BB is internalized to an endosomal compartment. TRAF2, and its K63 polyubiquitination activity, colocalizes with 4-1BB in endosomes. The TRAF2-associated E3 ubiquitin ligase activity and K63 polyubiquitination are required for the 4-1BB-mediated activation of the classical NF-κB pathway (116). Additionally, TRAF2 mediates the p38 MAP kinase pathway downstream of 4-1BB as T cells expressing a dominant negative form of TRAF2 lose the ability to signal via the p38 cascade (117).

### Coinhibitory signaling

#### CTLA-4 signaling

The inhibitory receptor CTLA-4 is constitutively expressed on Tregs and highly expressed on T_FR_ cells (118). CTLA-4 plays a key role in the suppressive functions of Tregs (119) and *Ctla4*^−/−^ mice develop systemic immune dysregulation, including increased T_FH_ and GC B cell responses (58). Short-term blockade with anti-CTLA-4 Ab or Treg-specific deletion of CTLA-4 increases T_FH_ and GC B cell responses *in vivo* (58, 120, 121) and reduces the ability of T_FR_ cells to inhibit B cell activation *in vitro* upon coculture with T_FH_ cells (120). CTLA-4 expressed by Tregs/T_FR_ cells therefore has a major influence on T_FH_ responses. Similar to the findings in mice, heterozygous, deleterious mutations in the human *CTLA4* gene manifest an immune dysregulation disorder, characterized by lymphocytic infiltration of multiple non-lymphoid organs. These individuals exhibit increased frequency of circulating CXCR5^±^PD1^±^ T_FH_ cells, which is normalized in response to treatment with CTLA4-Ig therapy (122).

CTLA-4 is also expressed by T_FH_ cells, although at a lower level than in T_FR_ cells (120). The cell-intrinsic role of CTLA-4 in T_FH_ differentiation and functions is far less defined than its cell-extrinsic role through Tregs and T_FR_ cells. One study reported that late deletion of CTLA-4 from *in vivo* differentiated T_FH_ cells using an inducible Cre/lox system increased their ability to induce isotype class switching and IgG production upon coculture with B cells (120). T_FH_-expressed CTLA-4 might therefore function to limit the B cell-stimulating activity of T_FH_ cells in a cell-intrinsic manner. Its contribution to T_FH_ differentiation during interactions between nascent T_FH_ cells and B cells is currently unknown.

CTLA-4 delivers its negative signaling via multiple mechanisms. At the cell surface, CTLA-4 competes with CD28 for access to the CD80/86 ligands. Through a process called trogocytosis, CTLA-4 removes CD80/86 ligands from the surface of APCs, further limiting the availability of these ligands for CD28 (123). Intracellularly, the tyrosine-phosphorylated cytoplasmic domain of CTLA-4 can interact with the phosphatases SHP-2 and PP2A (124, 125). Altogether, these CTLA-4-mediated cell-extrinsic and cell-intrinsic mechanisms dampen signaling downstream of the TCR and CD28. As such, it is conceivable that TRAF molecules, which can modulate TCR and CD28 signaling (see above), may potentially influence the CTLA-4-mediated regulatory pathway to modulate T cell signaling during an immune response. On the other hand, in Foxp3^+^ Tregs, CTLA-4 recruits the kinase PKCη to potentiate its suppressive functions *in vitro* and *in vivo* (126, 127). The CTLA-4-PKCη complex promotes the activation of the canonical NF-κB pathway in Tregs, representing a unique positive signaling event (126). It remains, however, to be determined whether and how the CTLA-4-PKCη axis regulates the activity of the T_FR_ subset. Although two other members of the novel PKC family, PKCδ and PKCε, have been shown to promote TRAF2 phosphorylation, IKK, and NF-κB activation in response to TNFα (128), it is unknown whether TRAFs are involved in the CTLA4-PKCη signal transduction pathway.

#### PD-1 signaling

GC T_FH_ cells express high levels of PD-1, consistent with this immunomodulatory protein being upregulated following chronic TCR stimulation, such as in the case of persistent interaction between T and B cells, which occurs during T_FH_ differentiation. The PD-1 ligands, PD-L1, and PD-L2, are also highly expressed by GC B cells (129). PD-1 inhibits T cell activation by suppressing CD28 costimulatory signaling (130). In the absence of PD-1, early T_FH_ differentiation is not affected, but the GC T_FH_ cell population is enriched at later time points (129). Similar studies investigating the role of PD-1-PD-L1 interaction in T_FH_ responses consistently report an expansion of T_FH_ cells in *Pdl1*^−/−^ mice and PD-1-deficient (*Pdcd1*^−/−^) mice, respectively, following protein immunization and viral infection (131–133). These findings reveal that PD-1 signaling can limit the proliferation of T_FH_ cells (134).

Surprisingly, the absence of PD-1 signals leads to a reduction of B cell responses in some studies, despite an expansion of the T_FH_ cell population (129, 131, 133). In one study, the increased T_FH_ cell numbers observed in *Pdcd1*^−/−^ mice is associated with a reduced synthesis of *Il4* and *Il21* mRNA by these cells (129), potentially explaining the reduced GC B cell responses. The discrepancy between the increased T_FH_ cell numbers and the reduced B cell responses could also result, in part, from the contributions of PD-1 to the T_FR_ cell population. In one study, *Pdcd1*^−/−^ mice have elevated numbers of T_FR_ cells that display enhanced suppressive activity following immunization with NP-OVA (135). The contribution of this suppressive population has not been assessed in other studies. It is possible that PD-1 affects the ratio between T_FH_ and T_FR_ cells differently in various models.

The ability of PD-1 to inhibit T cell activation depends on the recruitment of phosphatases SHP-1 and SHP-2 to the cytoplasmic domain of PD-1 (136, 137). More importantly, CD28 costimulatory signaling is distinctively sensitive to the PD-1-associated phosphatase activity (130). The recruitment of p85α and the phosphorylation of the CD28-associated kinases, Lck and PKCθ, are attenuated by the PD-1-SHP complex (130, 136). Interestingly, TRAF6 interacts with SHP-1, and this molecular complex restrains the phosphorylation of the p85α subunit of PI3K and the activation of the canonical NF-κB pathway (138), suggesting that TRAF6 might interfere with the co-inhibitory signaling of PD-1.

#### BTLA signaling

BTLA, the ligand of the TNFRSF member HVEM, is highly expressed on T_FH_ cells. *Btla*^−/−^ mice have elevated level of IgG in response to the T-cell dependent NP-KLH antigen (139). Moreover, *Btla*^−/−^ mice produce autoantibodies spontaneously (140), indicating that BTLA acts as a negative regulator of the humoral response. Upon immunization, T_FH_ generation is not affected in *Btla*^−/−^ mice, but the number of GC B cells is elevated (141). BTLA acts in a T cell-intrinsic fashion as *Btla*^−/−^ CD4^+^ T cells activated *in vitro* in presence of IL-6 increase the production of IL-21, and promote IgG2a and IgG2b Ab responses upon *in vivo* transfer.

Similar to PD-1, BTLA relies on dual tyrosine phosphorylation motifs in its cytoplasmic tail to recruit SHP-1 and SHP-2 in T cells (142). Because TRAF6 interacts with SHP-1 (138), it is conceivable that TRAF6 might affect BTLA signaling.

### Cytokines and STAT signaling

In addition to the TCR and costimulatory receptors, interactions of autocrine or paracrine cytokines with their cognate receptors provide essential signals that regulate the differentiation and function of T_FH_ cells. The signal transducer and activator of transcription (STAT) proteins are critical integrators of cytokine signals. Multiple STAT molecules can be activated simultaneously by one or more cytokines (143). The differentiation of T_FH_ cells is positively or negatively modulated by STAT3-dependent cytokines (IL-6, IL-21, IL-27) and STAT5-dependent cytokines (IL-2 and IL-7), respectively. Interestingly, several TRAF molecules are involved in these cytokine/STAT signaling cascades.

#### IL-6

IL-6 is a pleiotropic cytokine that plays a major role in inflammation. Several studies have independently demonstrated the importance of IL-6 in T_FH_ generation (78, 144, 145). Indeed, *Il6*^−/−^ mice display reduced GC formation and humoral responses (146). An initial spike of IL-6 production is detected on days 1–3 in both acute and chronic LCMV infection models (145). At the T cell priming stage, conventional DC secrete large amounts of IL-6 upon activation. IL-6 can transiently induce the expression of the transcription factor Bcl6 and cytokine IL-21 (42, 78), creating a positive feedback loop for enforcing the T_FH_ cell fate. Hence, the early programming of T_FH_ cells is abated in the absence of IL-6 (144). However, during chronic infection with LCMV Clone 13, a second wave of IL-6 expression is observed 3 weeks post-infection. FDC are responsible for the production of IL-6 at this late phase of viral infection (145). Administration of an IL-6-neutralizing Ab or IL-6R-blocking Ab 20 days after infection reduces *Bcl6* expression, T_FH_ and GC B cells (145). Interference with IL-6 functions also impairs the host's ability to clear the virus, indicating a late, but critical, role of IL-6 in maintaining an intact humoral response. However, other studies demonstrate that the differentiation of T_FH_ cells is not compromised in *Il6*^−/−^ mice or upon IL-6 neutralization (147–149), indicating that other signals, including IL-21, may compensate for the absence of IL-6 (see below).

Interestingly, the impact of T cell-specific deletion of IL-6Rα is less profound than the systemic deletion of IL-6 (144, 149, 150). The proportion of CXCR5^+^PD-1^+^ T_FH_ cells is moderately reduced in *Il6ra*^fl/fl^ *Cd4*^*Cre*^ mice following antigen immunization. Antigen-specific T_FH_ cells generated *in vivo* in the absence of IL-6Rα show reduced expression of Bcl-6 and IL-21 (150). However, there is a significant reduction in the fraction of GC B cells and plasma cells, implying that IL-6Rα signaling is indispensable for T_FH_ cell functions. At the molecular level, the IL-6 receptor is composed of IL-6Rα and the glycoprotein 130 (gp130), a signal transducer common to IL-6 receptor family members. TRAF2 and TRAF5 constitutively associate with gp130 (35, 36). This interaction suppresses the recruitment of STAT3 to the IL-6R complex, because they compete for the same binding site on gp130 (35, 36). Therefore, TRAF2 and TRAF5 are negative regulators of the IL-6R signaling pathway that could potentially limit the induction and functions of T_FH_ cells (Figure 1).

#### IL-21

IL-21 is a member of the common γ-chain family of cytokines produced by activated T and B cells. Its cognate receptor, IL-21R, is also highly expressed on T_FH_ cells (151) and GC B cells (152). Interestingly, lack of IL-21 or IL-21R does not affect the initial differentiation and expansion of T_FH_ cells (153, 154). However, the contraction of CXCR5^+^ PD-1^+^ GC T_FH_ cells occurs at a faster rate in *Il21*^−/−^ or *Il21r*^−/−^ mice after the first week of antigen challenge (153, 154). Although T cells are found in the GCs, these T cells are not able to support GC reactions in *Il21*^−/−^ or *Il21r*^−/−^ mice, leading to diminished levels of GC B cells, plasma cells, and serum IgG. Taken together, these data suggest that the IL-21-IL-21R axis is required for the T_FH_ cell persistence and functions.

Despite supportive evidence, the T-cell intrinsic role of IL-21 in the generation of humoral responses is hotly contested. Studies using *Il21*^−/−^ and *Il21r*^−/−^ mice show that the *in vivo* generation of CXCR5^+^PD-1^+^ T_FH_ cells in these mice is as robust as in wt mice following NP-KLH (152) or NP-CGG (147) immunization, or infection with LCMV (148) or Influenza (149). Several additional studies provide potential insights into this discrepancy: First, while the loss of either IL-6 or IL-21 alone has only a marginal effect on T_FH_ development and GC formation in response to acute viral infection, the simultaneous loss of both cytokines in *Il6*^−/−^*Il21*^−/−^ mice (149) or the neutralization of IL-6 in *Il21*^−/−^ mice (148), significantly blunts the antiviral Ab responses. These results indicate that IL-6 and IL-21 can act redundantly or complementarily to promote T_FH_ development. This is mechanistically conceivable because IL-6 and IL-21 signal predominantly through the same intracellular signal transducer, STAT3 (see below). Second, even in the presence of an intact T_FH_ cell population, *Il21*^−/−^ and *Il21r*^−/−^ mice are severely defective in mounting Ab responses. Mixed bone marrow chimera experiments revealed that IL-21 acts directly on B cells (152). In the absence of IL-21, the proliferation of GC B cells is significantly curtailed at the later stage of viral infection. However, as mentioned earlier, T_FH_ and B cells in GCs are mutually dependent on each other. The absence of either IL-21 or IL-21R on either T or B cells could lead to similar defects. Therefore, the impaired T cell-dependent humoral responses in intact *Il21*^−/−^ and *Il21r*^−/−^ mice do not reveal whether IL-21 acts in an autocrine fashion or, alternatively, whether T and/or B cells respond to IL-21 in a paracrine fashion. Transfer of wt or *Il21r*^−/−^ T cells and B cells into irradiated recipient mice shows that the presence of IL-21R on both T and B cells is required for the optimal production of high-affinity Abs in response to LCMV infection (154). Nonetheless, owing to the essential and yet complicated roles of IL-21 in T-dependent humoral responses, a “cleaner” experimental setup, based on the inducible Cre/lox system, would be required to rigorously dissect the spatiotemporal functions of IL-21 and/or IL-21R in T and B cells.

TRAF5 acts a negative regulator of IL-21 production. *Traf5*^−/−^ CD4^+^ T cells secrete significantly elevated amount of IL-21 upon CD3 plus CD28 costimulation in the presence of IL-6 and TGF-β (35). This effect is dependent on the presence of IL-6 as the binding of TRAF5 to the IL-6R complex restricts the activation of STAT3 (Figure 1).

#### IL-27

IL-27 is a member of the IL-6/IL-12 family of cytokines, which binds to a heterodimeric receptor consisting of IL-27Rα and gp130 subunits. *Il27ra*^−/−^ mice display defective development of CXCR5^+^PD-1^+^ GC T_FH_ cells (155). Stimulation with recombinant IL-27 *in vitro* enhances ICOS expression and IL-21 production from naïve CD4^+^ T cells (155, 156). Additionally, IL-27 is required to promote the maturation of GC B cells (157). *In vivo*, IL-27 promotes T-dependent Ab responses through a combination of T- and B cell-intrinsic mechanisms (157). Because IL-6 and IL-27 share the gp130 subunit, it is possible that TRAF2 and TRAF5 could similarly modulate the signaling events downstream of IL-27R, and hence, alter the differentiation and functions of T_FH_ cells.

#### Type I IFN

The dependence on STAT1 for the early stage of T_FH_ differentiation (144) implies a role for type I IFNs (IFNα/β) in this process, because STAT1 is the key transcription regulator downstream of the type I IFN signaling pathway. IFNα/β are ubiquitous cytokines produced by innate immune cells during the early phase of viral infection. An early report demonstrates that exogenous administration of IFNα/β strongly promotes the production of IgG in a dose-dependent manner following antigen immunization (158). Conversely, in the absence of the IFNα/β receptor (IFNAR) subunit IFNAR1, the differentiation of T_FH_ cells, migration of T_FH_ cells into the GC, and B cell responses are impaired following immunization (159–161). Mechanistically, IFNα/β signaling in DC induces the production of IL-6, which in turn promotes T_FH_ differentiation *in vivo* (159). *In vitro* treatment of CD4 T cells with IFNα/β induces the expression of Bcl6, CXCR5 and PD-1, but not the production of IL-21, suggesting that T cell-intrinsic IFNα/β signaling can positively contribute to the T_FH_ differentiation. In agreement, in mixed bone marrow chimera experiments, the T_FH_ differentiation of *Ifnar*^−/−^ T cells is compromised, compared to wt T cells in the same recipients, demonstrating a T cell-intrinsic role of IFNα/β in the T_FH_ differentiation following immunization (160, 161). Paradoxically, in the context of experimental *Plasmodium* infection, the differentiation of T_FH_ cells, GC B cells, and Ab responses are significantly enhanced in *Ifnar1*^−/−^ mice or upon anti-IFNAR1 Ab neutralization (162, 163), implying a negative role of IFNα/β signaling in parasitic infections. The contrasting roles of IFNα/β in the differentiation and functions of T_FH_ cells might reflect the differential requirement of IFNα/β and its signaling in viral *vs*. parasitic infections.

Upon stimulation with IFNβ, TRAF2 coimmunoprecipitates with the IFNAR1 subunit of the IFN receptor complex (164). Analysis of *Traf2*^−/−^ mouse embryonic fibroblasts (MEF) shows that the formation of the p52-p65 complex in the non-canonical NF-κB signaling pathway is absent upon stimulation with IFNβ. However, the IFN-induced activation of the canonical NF-κB pathway, and the phosphorylation of STAT1, STAT2, and STAT3 are indistinguishable between WT and *Traf2*^−/−^ MEFs. Therefore, in lieu of TRAF2, other TRAF molecules might regulate these latter signaling cascades in response to IFNα stimulation (see below).

#### STAT1 and STAT3

As described above, IL-6,−21, and−27 promote the differentiation, persistence and functions of T_FH_ cells in a T cell-intrinsic manner. A common feature among these T_FH_-inducing cytokines is their signaling via the transcription factors STAT1 and STAT3. Not surprisingly, STAT1 or STAT3 deficiency affects the generation of T-dependent B cell memory and high affinity Ab-secreting cells. The lack of STAT3 leads to profound defects in the acquisition of B cell help functions. T-cell specific deletion of STAT3 significantly impairs the number of CXCR5^+^ T_FH_ cells, GC B cells, and IgG levels in mice following challenge with antigen plus adjuvant or LCMV infection (78, 165, 166). In humans, individuals with missense mutations or short deletions of *STAT3* suffer from Hyper-IgE syndrome, a primary immunodeficiency characterized by heightened susceptibility to *Staphylococcus aureus* and *Candida albicans*. T cells from these STAT3 mutated individuals fail to upregulate IL-21 and provide help to B cells upon *in vitro* culture and a reduction of circulating CXCR5^±^ CD4^±^ T cells is observed in patients suffering from hyper IgE syndrome resulting from STAT3 mutations (167).

In addition, type I interferon also mediates its signaling through STAT1. Knockdown of STAT1 in mouse T cells results in defective generation of early CXCR5^+^Bcl6^+^ T_FH_ cells 2 days after infection. This defect is more pronounced when both STAT1 and STAT3 are absent, suggesting a redundant role of these transcriptional regulators as downstream mediators of IL-6, −21, −27, and type I interferon (144).

The crosstalk between TRAF proteins and STATs has only been studied in recent years. As aforementioned, gp130, which mediates signaling downstream of IL-6 and IL-21, interacts with TRAF2 and TRAF5. *Traf5*^−/−^ CD4^+^ T cells exhibit an elevated phosphorylation of JAK1 kinase upon stimulation with IL-6, suggesting that the recruitment of these TRAFs to the IL-6R and IL-21R complexes limits the phosphorylation of JAK1 in T cells (168).

In addition, TRAF6 associates with, and mediates the ubiquitination of STAT3 in fibroblasts (169). This interaction represses the transcriptional activity of STAT3 and downregulates the expression of STAT3-regulated genes upon stimulation with IFNα (169), suggesting that TRAF6 acts as a negative signaling mediator of STAT3 downstream of Type I interferon signaling. TRAF3 inhibits STAT3 activation downstream of IL-6R signaling in B cells (170). Moreover, TRAF3 is required for the association of the phosphatase PTPN22 with JAK1, which in turn inhibits STAT3 phosphorylation (170). TRAF3 and−6 might negatively regulate STAT3 activity in T cells via similar mechanisms.

#### IL-12

IL-12 is well known for its key role in inducing Th1 differentiation in both mouse and humans. However, in rodents, stimulation of naïve mouse CD4 T cells *in vitro* in the presence of IL-12 induces the expression of both the T_FH_ transcription factor Bcl6 and the Th1 transcription factor T-bet (171). IL-21^±^, IFNγ^±^, and IL-21^±^ IFNγ^±^ cells are simultaneously present in the *in vitro* culture. However, the percentage of IL-21 producing cells declines rapidly over time in favor of IFNγ^±^ cells, coinciding with reduction of Bcl6 expression in favor of T-bet (171).

IL-12 has been shown to mediate the differentiation of human T_FH_ cells. Activated DC can induce naïve human CD4 T cells to produce IL-21 in an IL-12 dependent manner (172), and conversely, naïve human T cells primed with IL-12 can induce B cells to produce Ig *in vitro* (172). *In vitro* stimulation of naïve human CD4 T cells in the presence of IL-12 also induces the expression of CXCR5, Bcl6, and ICOS (172–174). The role of IL-12 signals for T_FH_ generation is also important for *in vivo* responses as individuals deficient in the IL12-receptor subunit IL-12Rβ1 display less circulating CXCR5^±^ CD4 T cells, altered GC responses, and reduced numbers of memory B cells (174). Induction of IL-21 and Bcl6 by IL-12 depends on the transcription factor STAT4 (171, 172). GC T_FH_ in human tonsils show high levels of activated STAT4, suggesting that they could be actively receiving IL-12 signals *in vivo* (174). Concomitantly, the generation of T_FH_ and GC B cells is impaired in *Stat4*^−/−^ mice 4 days following immunization, but not at later stages (171). To date, no TRAF activity has been identified in the IL-12R or STAT4 signaling.

#### IL-2

IL-2 acts primarily on T cells via the IL-2R, consisting of the α, β, and the common γ subunits. The high-affinity IL-2Rα, CD25, is differentially expressed in T_FH_ and non-T_FH_ cells. CD25 is downregulated in Bcl6^+^CXCR5^+^ T_FH_ cells, whereas CD25^+^ T cells express the transcription factor Blimp1, which is antagonistic to Bcl6 (75). These findings support the notion that T_FH_ cells do not require IL-2 signaling for their differentiation and functions. In fact, the expression of Bcl6 is elevated under limiting IL-2 conditions. The accumulated Bcl6 proteins in turn bind to DNA and repress its direct target *Prdm1* (which encodes the transcriptional repressor Blimp-1) (175). Reduction of IL-2 signaling results in increased T_FH_ cell differentiation during the early DC priming phase in *Il2ra*^+/−−^ mice (176) or upon anti-IL-2 Ab-mediated neutralization. Correspondingly, treatment with recombinant IL-2 impairs T_FH_ differentiation and suppresses GC B cell responses (177).

TRAF3 and 6 are both negative regulators of IL-2 signaling (Figure 1). TRAF3 is recruited to the IL-2 receptor complex and promotes the recruitment of the phosphatase TCPTP. *Traf3*^fl/fl^ *Cd4*^Cre^ T cells show enhanced phosphorylation of Jak1, Jak3, and STAT5 upon IL-2 stimulation (33). Additionally, TRAF6 coimmunoprecipitates with IL-2Rβ in 293T cells coexpressing those two proteins, and preactivated *Traf6*^−/−^ CD4^+^ T cells display enhanced phosphorylation and activation of Jak1 and Erk in response to IL-2 (34). TRAF3 and TRAF6 could therefore contribute to the control of T_FH_ differentiation by modulating IL-2 signals.

#### IL-7

IL-7, a member of the IL-2 cytokine family, is important for T and B cell survival, proliferation and development. Similar to CD25, IL-7Rα expression is strongly downregulated during T_FH_ differentiation, as early as 3 days following LCMV infection (178). IL-7Rα is then progressively reexpressed and GC T_FH_ cells express high IL-7Rα levels (178), consistent with a role for IL-7 in the long-term survival of memory T cells. The early downregulation of IL-7Rα suggests a negative role for IL-7 signals in the differentiation of T_FH_ cells. Indeed, Bcl6 represses IL-7R (179) and, reciprocally, T_FH_ exposure to IL-7 represses the expression of the key T_FH_ genes, *Bcl6* and *Cxcr5* (180). Consistent with these findings, administration of anti-IL7Rα Ab enhances T_FH_ development and GC reactions, whereas transgenic expression of IL-7Rα by CD4^+^ T cells reduces their T_FH_ differentiation (179). Intriguingly, one study showed the opposite, i.e., positive role of IL-7 on T_FH_ cells. The administration of exogenous Fc-fused IL-7 significantly increases both CD4^+^ and CD8^+^ T cell responses induced by a DNA vaccine (181). The enhancement of CD4^+^ T cell responses was accompanied by the expansion of T_FH_ cells, GC B cells, and GC reactions (181). The enhanced development of T_FH_ cells in this experimental model is not dramatically affected by IL-6 and IL-21 neutralization, suggesting an independent role of IL-7 in T_FH_ differentiation. No TRAF activity has been associated with IL-7R.

#### STAT5

IL-2 and IL-7, which negatively regulate T_FH_ generation, signal through STAT5. STAT5 acts as a transcriptional repressor for the expression of Bcl6 (182). Inhibition of Bcl6 expression correlates with the enhanced binding of STAT5 to the *Bcl6* promoter region in Th1 cells stimulated with IL-2 *in vitro* (175). Similarly, in IL-7 stimulated cells, there is an increase of STAT5 binding to the *Bcl6* gene promoter, leading to a reduction in Bcl6 expression (180). Accordingly, T cell-specific deletion of STAT5 increases T_FH_ cell development, GC B cell numbers, and Ab levels following immunization (183). On the other hand, the presence of a constitutively active STAT5 mutant in antigen-specific T cells blocks the differentiation of T_FH_ cells following LCMV infection (176).

TRAF3 and 6 are recruited to the IL-2R and negatively regulate its signaling activity (33, 34). After IL-2 stimulation, activation of STAT5 is enhanced in *Traf3*^fl/fl^ *Cd4*^Cre^ T cells, suggesting that TRAF3 acts as a negative regulator of STAT5 (33).

#### TGF-β

In the human immune system, TGF-β alone is insufficient to induce expression of the T_FH_ cell phenotype (184). *In vitro* TGF-β stimulation in combination with IL-12 or IL-23 optimally promotes the expression of T_FH_-associated molecules Bcl6, CXCR5, ICOS and IL-21, and antagonizes Blimp1 expression, in naïve human CD4^+^ T cells (184). Elevated phosphorylation of Smad2, a downstream effector of TGF-β signaling, is found in T cells localized close to the GC in tonsils, suggesting that TGF-β signaling is likely to participate in human T_FH_ differentiation (184). However, the requirement of TGF-β for T_FH_ differentiation appears to be species-specific as *in vitro* stimulation of murine T cells with TGF-β inhibits the induction of Bcl6, IL-21 and ICOS (42, 184, 185). In contrast, experiments using adoptive transfer of antigen-specific *Tgfbr2*^−/−^ T cells revealed that T cell-intrinsic TGF-β signaling is required for the differentiation of CXCR5^+^PD-1^+^ T_FH_ cells and the generation of GC B cell and Ab responses *in vivo* following LCMV infection (186). TGF-β suppresses the expression of CD25. The absence of IL-2 signaling, in turn, is beneficial for the early induction of T_FH_ cells.

*In vitro* stimulation of *Traf6*^fl/fl^ *Cd4*^cre^ murine T cells in the presence of TGF-β shows enhanced and sustained Smad2 and Smad3 phosphorylation. This sustained TGF-β signaling results in lower *Il2* mRNA and protein levels (187). Therefore, TRAF6 acts as a negative regulator of Smad-mediated TGF-β signaling in T cells, and thus, may influence the differentiation and functions of T_FH_ cells (Figure 1).

## TRAF-mediated canonical and non-canonical NF-κB signaling in T_FH_ cell differentiation

**Figure 2:**
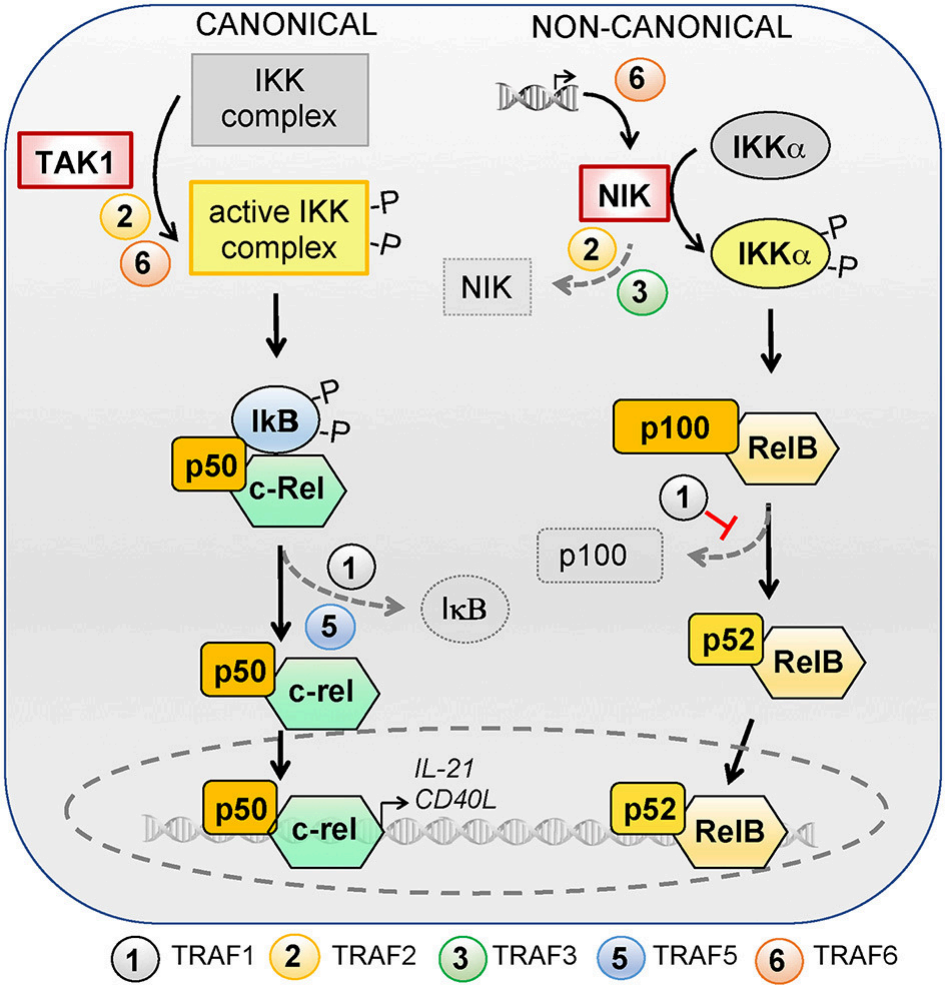
Role of TRAFs in the canonical and non-canonical NF-κB signaling pathways. NF-κB can be activated via the canonical or non-canonical signaling pathways. The canonical pathway is controlled by TAK1 kinase activation, which activates the IKK complex and leads to ubiquitylation and proteasomal degradation of IκB family members, resulting in the release and nuclear translocation of NF-kB1/p50–RelA/p65 and NF-κB1/p50–c-Rel dimers. RelA/p65 is dispensable for T_FH_ differentiation but c-Rel regulates the expression of IL-21 and CD40L and is required for T_FH_ differentiation. TRAF2 and−6 favor IKK complex activation by TAK1 and TRAF1 and−5 are required for optimal IκB degradation. The activation of the non-canonical NF-κB pathway depends on the NF-κB-inducing kinase NIK. NIK can phosphorylate and activate IKKα, which in turn promotes p100 processing to generate NF-κB2/p52 and allows nuclear translocation of NF-κB2/p52 and RelB. In the absence of activating signals, constitutive ubiquitylation and degradation of NIK ensures repression of the non-canonical NF-κB pathway. NIK deficiency in T cells does not impact T_FH_ differentiation. TRAFs regulate the non-canonical NF-κB pathway by modulating NIK expression levels: TRAF6 is involved in transcriptional regulation of *Nik* whereas TRAF2 and−3 contribute to its degradation. TRAF1 restrains the non-canonical NF-κB pathway activation by inhibiting p100 processing.

TRAF family members are critical signal transducers that relay signals between stimulus-sensing surface receptors and transcription regulators, ultimately leading to a change in gene expression. Many studies using different cell types and stimuli reveal that TRAF family members are involved in the activation of the transcription factors of the NF-κB family. NF-κB can be activated via two major pathways: the canonical and non-canonical signaling pathways [reviewed in (188, 189)]. Briefly, the canonical NF-κB pathway is controlled by TAK1 kinase activation that leads to the ubiquitination and proteasomal degradation of IκB family members, resulting in the release and nuclear translocation of the NF-κB1/p50–RelA/p65 and NF-κB1/p50–c-Rel dimers. On the other hand, activation of the non-canonical NF-κB pathway depends on the NF-κB-inducing kinase NIK. NIK can phosphorylate and activate IKKα, which in turn promotes p100 processing to generate NF-κB2/p52 and allow its nuclear translocation together with RelB. In the absence of activating signals, constitutive ubiquitination and degradation of NIK ensures the repression of the non-canonical NF-κB pathway. Herein, we will focus on the role of TRAF proteins in the canonical and non-canonical NF-κB signaling pathways (summarized in Figure 2) and discuss how TRAF-mediated NF-κB signaling can contribute to T_FH_ differentiation and T-dependent humoral responses. Readers are advised to refer to other chapters in this volume to gain a broader perspective of TRAF-mediated canonical and non-canonical NF-κB pathways in the immune system.

### TRAF-mediated canonical NF-κB signaling in T_FH_ cells

Several studies demonstrate the T cell-intrinsic requirement for canonical NF-κB signaling in T_FH_ differentiation. First, genetic ablation of the transcriptional subunit, NF-κB1/p50, in OT-II CD4^+^ T cells selectively impairs the upregulation of CXCR5 following immunization, leading to a severe defect in the generation of CXCR5^+^PD1^+^ GC T_FH_ cells and GC B cell responses (190). Second, because the NF-κB1/p50 subunit dimerizes with RelA/p65 or c-Rel, *Rel*^−/−^ mice (deficient for c-Rel) display defects in T cell-dependent humoral immunity (191). Subsequently, it was demonstrated that the mRNA and protein levels of IL-21 are reduced in *Rel*^−/−^ T cells, indicating that c-Rel positively regulates the expression of IL-21 in T cells (192). Moreover, the expression of c-Rel is regulated by a microRNA, miR-155 (193). T cell-specific ablation of miR-155 promotes the degradation of c-Rel, which impedes the upregulation of CD40L in *mir155*^−/−^ T cells, and severely impairs T_FH_ differentiation and B cell Ab responses *in vivo* (193). This defect can be restored by over-expression of c-Rel in *mir155*^−/−^ T cells, pointing to a T cell-intrinsic role for c-Rel in the control of T_FH_ differentiation. Interestingly, the other NF-κB1/p50 partner, RelA/p65, is dispensable for T_FH_ differentiation (194).

TRAF1, 2, 5, and 6 can positively regulate the activation of the canonical NF-κB pathway (Figure 2). *Traf1*^−/−^ T cells show reduced IκB degradation upon stimulation with anti-4-1BB Ab (23). TRAF2 knockdown impairs the canonical NF-κB activation induced by anti-CD3/CD28 stimulation in Jurkat T cells (195), by anti-OX40 stimulation in T cell hybridomas (101), or by anti-4-1BB in HEK293T fibroblasts (116). Preactivated *Traf5*^−/−^ T cells also show reduced canonical NF-κB activation upon treatment with anti-GITR Ab (104). In addition, knockdown of TRAF6 hinders the activation of the canonical NF-κB pathway in Jurkat T cells stimulated with anti-CD3/CD28 Ab (195), and the degradation of IκB is delayed in *Traf6*^−/−^ T cells (27). Hence, TRAF1, 2, 5, and 6 can contribute to T_FH_ differentiation by positively regulating the canonical NF-κB pathway.

### TRAF-mediated non-canonical NF-κB signaling in T_FH_ cells

The role of non-canonical NF-κB signaling in T cell-dependent Ab responses has been extensively studied in *Nik*^−/−^ mice, which display an impaired development of CXCR5^+^ PD-1^+^ GC T_FH_ cells (196). However, T_FH_ differentiation was not affected when NIK deficiency was restricted to T cells using an adoptive transfer model, implying that the role of NIK in T_FH_ differentiation is not T cell-intrinsic (196). Instead, the expression of NIK in B cells is required for the optimal expression of ICOSL, and, thus, the promotion of T_FH_ differentiation. These findings suggest that the non-canonical NF-κB signaling pathway in B cells, but not in T cells, is required for humoral responses. Although the deletion of either RelB or NF-κB2/p52 does not affect B cell responses, genetic ablation of both RelB and NF-κB2/p52 in GC B cells dramatically impedes GC reactions (197). Therefore, the non-canonical NF-κB signaling is more important in B cells, which in turn could affect the differentiation and maintenance of GC T_FH_ cells.

TRAF2 and TRAF3 play a negative role in the control of the non-canonical NF-κB pathway (Figure 2). The absence of either TRAF2 or TRAF3 results in the constitutive activation of this pathway in T cells (25, 198). TRAF2 and TRAF3 form a complex with cIAP1 and cIAP2, which are E3 ubiquitin ligases responsible for NIK ubiquitination and degradation. In the absence of TRAF2 or TRAF3, the cIAP-TRAF complex is disrupted, allowing an increase of NIK protein level and aberrant activation of the non-canonical NF-κB pathway (199–201). In a similar fashion, TRAF1 has also been found to restrain the non-canonical NF-κB pathway in response to stimulation with anti-CD3 Ab (23). On the other hand, TRAF6 acts as a positive regulator by inducting the expression of NIK, resulting in activation of the non-canonical NF-κB pathway in the presence of OX40 ligation (202).

As T cell-specific *Nik* deficiency did not affect T_FH_ differentiation (196), modulation of the non-canonical NF-κB pathway by TRAF2, 3, and 6 is not likely to directly impact on T_FH_ differentiation. However, because the non-canonical NF-κB pathway is important for B cell maturation, which in turn is required to maintain T_FH_ cells, TRAF1, 2, 3, and 6 might contribute to the overall T-dependent and T-independent Ab responses.

## T cell- and B cell-intrinsic roles of TRAFs in humoral responses

In this part, we will review the contribution of individual TRAF proteins to the development of T cell-dependent humoral responses and discuss whether each TRAF member influences humoral responses through T cell-intrinsic or B cell-intrinsic pathways. In humans, single nucleotide polymorphisms of several members of the TRAF family are associated with the development of SLE and RA (16), two autoimmune disorders with excessive T_FH_ responses and GC reactions (7). However, the mechanisms by which TRAFs contribute to disease susceptibility or development are unknown. In this part we will infer the potential contributions of each TRAF family member to the differentiation of T_FH_ cells at the mechanistic level, in light of the known role of TRAFs in the signaling pathways controlling T_FH_ differentiation reviewed in the preceding sections.

### TRAF1

*Traf1*^−/−^ mice display normal T cell and B cell lymphocyte development (22). Increased T cell proliferation of *Traf1*^−/−^ T cells is observed in response to anti-CD3 Ab (22, 23) or antigen stimulation (203). *Traf1*^−/−^ CD4^+^ T cells express higher levels of the Th2 cytokines IL-4, IL-5, and IL-13 upon *in vitro* stimulation. Accordingly, transfer of OVA-stimulated *Traf1*^−/−^ CD4^+^ T cells into naïve wt recipients trigger an enhanced asthmatic response following aerosol inhalation with ovalbumin as compared to the transfer of OVA-stimulated wt CD4^+^ T cells (203). TRAF1 has been reported to associate with CD40 (204). However, the proliferation of *Traf1*^−/−^ B cells is not affected upon *in vitro* stimulation with anti-IgM or anti-CD40 Abs, or *in vivo* challenge with T cell-independent antigens (22). *Traf1*^−/−^ mice display normal IgG1, IgG2a, and IgE anti-ovalbumin responses, suggestive of an intact B cell isotype switching and T cell help (22). These data suggest that TRAF1 is dispensable for the development of T cell-dependent humoral responses.

### TRAF2

*Traf2*^−/−^ mice are embryonic lethal as a result of excessive TNFα production. Simultaneous deletion of the TNFα-TNFR1 axis results in partial rescue of the *Traf2*^−/−^*Tnf*^−/−^ or *Traf2*^−/−^*Tnfr1*^−/−^ animals (205). *Traf2*^−/−^*Tnf*^−/−^ mice display normal IgM levels in response to VSV infection, but the IgG responses are abrogated. B cells from *Traf2*^−/−^*Tnfr1*^−/−^ mice fail to proliferate and activate NF-κB in response to *in vitro* anti*-*CD40 stimulation (205). These data are consistent with the fact that TRAF2 interacts with CD40 (204), and that this interaction is essential for isotype switching (206). However, the fact that these models lack TRAF2 and TNFα-TNFR1 signaling confounds the interpretation regarding the actual role of TRAF2 in the development of T cell-dependent Ab responses.

To better define the roles of TRAF2 in B cells, *Traf2*^fl/fl^ *Mx1*^Cre^ and *Traf2*^fl/fl^ *Cd19*^Cre^ mice were generated, resulting in B cell-specific TRAF2 deletion (198, 207). Unexpectedly, B cell specific TRAF2 deficiency resulted in increased B cell numbers in the secondary lymphoid organs. *Traf2*^−/−^ B cells display enhanced survival, increased cell size and constitutive activation of the non-canonical NF-κB pathway. Nevertheless, the CD40-mediated activation of the canonical NF-κB pathway and B cell proliferation are impaired in the absence of TRAF2, implying a positive regulatory role of TRAF2 in CD40 signaling. Similar to the *Traf2*^fl/fl^ *Mx1*^Cre^ and *Traf2*^fl/fl^ *Cd19*^Cre^ mice, mice expressing a TRAF2 dominant negative transgene (TRAF2-DN) devoid of the N-terminal RING and zinc finger domains display splenomegaly and lymphadenopathy (208). Surprisingly, the canonical NF-κB pathway is unperturbed in B cells isolated from TRAF2-DN mice in response to CD40L or TNFα stimulation. Instead, the activation of the JNK pathway is dependent on TRAF2 following CD40L or TNFα stimulation (208). The functional discrepancy between *Traf2*^−/−^ and TRAF2-DN-expressing B cells could be explained by the conservation of TRAF2-mediated protein-protein interactions in TRAF2-DN-expressing B cells.

The contribution of TRAF2 to T cell functions and its implication in the regulation of Ab responses is under-explored. T cells from TRAF2-DN (208, 209) and *Traf2*^fl/fl^ *Lck*^Cre^ mice (24) show defective *in vitro* T cell proliferation in response to anti-TCR stimulation or allogenic APCs. *Traf2*^−/−^ T cells show a propensity to skew into the Th2 lineage upon *in vitro* polarization and their Th17 differentiation is impaired (24). This is associated with reduced JNK and canonical NF-κB pathway activation following stimulation with TNFα. To date, a T cell-intrinsic role for TRAF2 in T_FH_ differentiation has not been reported. However, because TRAF2 is involved in recruitment of PI3K to OX40 (210) and activation of the classical NF-κB pathway downstream of OX40 (101) and GITR (103), two key molecules promoting T_FH_ differentiation, TRAF2 could potentially play a positive regulatory role in the differentiation of T_FH_ cells. On the other hand, TRAF2 has been shown to restrain IL-6 signaling, suggestive of a negative role in T_FH_ differentiation [Figures 1, 2; (36)]. Therefore, the overall role of TRAF2 in T-dependent Ab responses awaits further exploration.

### TRAF3

*Traf3*^−/−^ mice die within 10 days of birth. To assess the contribution of TRAF3 to T cell-dependent Ab responses, fetal liver cells were used to reconstitute the hematopoietic system of sublethally irradiated recipients (211). *Traf3*^−/−^ fetal liver cells could reconstitute the T cell, B cell, granulocytic, and erythroid lineages, and reconstituted recipients survived longer than 6 months. Using this chimeric system, recipient mice reconstituted with *Traf3*^−/−^ cells failed to produce antigen-specific IgG in response to T cell-dependent antigens. However, the proliferation of *Traf3*^−/−^ B cells in response to stimuli such as anti-IgM Ab and CD40L was normal. Because the T cell recall response after *in vivo* immunization is dramatically reduced in the absence of TRAF3, it was concluded that TRAF3 is required for T cell help (211).

To circumvent the early postnatal lethality of *Traf3*^−/−^ mice, *Traf3*^fl/fl^ *Cd19*^Cre^ mice with B cell-specific TRAF3 deletion were generated (198, 212). These mice exhibited splenomegaly and lymphoadenopathy, with a concomitant elevation of follicular B cells, spontaneous GC formation, hyperimmunoglobulinemia, T cell-independent Ab responses, and exacerbated autoimmune manifestations (212). At the molecular level, *Traf3*^−/−^ B cells exhibit constitutive activation of the non-canonical NF-κB pathway, supporting the survival of B cells. In spite of all these B cell defects, the development of GC B cells in *Traf3*^fl/fl^ *Cd19*^Cre^ mice following immunization remained intact (213). Paradoxically, B cell-specific overexpression of TRAF3 in a transgenic mouse strain induced excessive systemic inflammation, autoimmunity, and hyperimmunoglobulinemia at an old age (214). These transgenic mice are hyperresponsive to T-dependent and T-independent antigen challenges, despite the fact that the over-expression of TRAF3 does not alter the CD40-mediated NF-κB and MAP kinase pathways in B cells. Altogether, these results indicate an important, yet complicated, role of TRAF3 in regulating B cell homeostasis.

To understand the roles of TRAF3 in T cell biology, *Traf3*^fl/fl^ *Cd4*^Cre^ mice were generated. These *Traf3*^fl/fl^ *CD4*^Cre^ mice are born at the expected Mendelian ratio, and they survive and breed normally (25). Following immunization with a T-dependent antigen, the antigen-specific IgG1 Abs are nearly absent in these mice, indicative of a T cell-intrinsic role of TRAF3 (25). In a *Listeria monocytogenes* infection model, *Traf3*^fl/fl^ *Cd4*^Cre^ mice are much more sensitive to bacterial challenge, displaying a higher bacterial load and lower numbers of IFNγ-producing T cells in the liver, demonstrating that *Traf3*^−/−^ T cells are compromised (25). Mechanistically, TRAF3 is recruited to the TCR-CD28 complex and it participates in the activation of proximal TCR signaling (Figure 1). In its absence, proliferation and cytokine production are impaired in stimulated CD4^+^ and CD8^+^ T cells.

Interestingly, Treg-selective TRAF3 ablation in *Traf3*^fl/fl^ *Foxp3*^Cre^ mice leads to a marked reduction in T_FR_ cell induction following immunization, resulting in increased expression of *Bcl6, Cxcr5* and the cytokines genes *Il-4, Il-10, Il-17*, and *Ifng* by T_FH_ cells, coupled with sustained GC reactions and production of high-affinity IgG Abs (215). The expression of ICOS is reduced in *Traf3*^−/−^ T_FR_ cells because of the inactivation of TRAF3-dependent ERK and AP-1 signaling pathways (215). However, whether TRAF3 influences T_FH_ differentiation in a cell-intrinsic way remains to be determined. Interestingly, alternative splicing of TRAF3 can generates a TRAF3 isoform that mediates activation of the non-canonical NF-κB pathway and the production of CXCL13 by T cells (216). Although the relevance of TRAF3-controlled CXCL13 production *in vivo* in T cell-dependent Ab responses remains to be elucidated, these data suggest a positive role for TRAF3 in GC formation by favoring T_FH_ cells migration into the GC. TRAF3 might also positively influence T_FH_ differentiation by enhancing TCR- and CD28-induced signaling (25, 26) and restraining IL-2R signals [Figure 1; (33)]. Conversely, TRAF3 can inhibit IL-6R signaling in B cells (170) and negatively regulate OX40-induced NF-κB signaling in HEK293T cells (217). It is currently unknown whether a similar TRAF3-mediated regulation of IL-6R and NF-κB signaling occurs in primary T cells.

### TRAF4

*Traf4*^−/−^ mice show normal T and B cell differentiation. The T cell-dependent IgG response to OVA immunization is unaffected in *Traf4*^−/−^ mice (218). Although the T_FH_ cell population has not been investigated in this study, this finding suggests that TRAF4 is dispensable for the differentiation and functions of T_FH_ cells. No other studies to date have demonstrated a role for TRAF4 in primary T cell functions.

### TRAF5

*Traf5*^−/−^ mice show unaltered development of T and B cell lineages (219, 220). *Traf5*^−/−^ mice produce similar titers of IgG1 Ab than wt controls following antigen immunization, but there is a slight reduction in Ab affinity maturation (219). *Traf5*^−/−^ T cells produce increased amounts of IL-4 and IL-5 in response to OX40 stimulation *in vitro*, and develop a more severe Th2-driven allergic lung inflammation following antigen immunization and airway challenge (220). In this model, *Traf5*^−/−^ mice produce enhanced levels of OVA-specific IgE. Altogether, these data suggest that the T cell-dependent class switching and production of Ab *in vivo* are not dramatically affected by TRAF5 deficiency.

Notably, *Traf5*^−/−^ T cells display impaired GITR signaling, with decreased canonical NF-κB, Erk, and p38 activation, and exhibit reduced proliferation and IL-2 production upon stimulation in presence of anti-GITR Ab (104). TRAF5 constitutively associates with the gp130 subunit of the IL-6R and negatively regulates IL-6R signaling by suppressing the recruitment of STAT3 to the IL-6R complex (35, 36). The role of TRAF5 in the development and maintenance of T_FH_ responses *in vivo* remains to be investigated. Because GITR signaling (19), activation of the canonical NF-κB pathway (190, 191), and IL-6 signaling (78, 144, 145) are all important for T_FH_ development, one could predict that TRAF5 is also involved in modulation of T_FH_ differentiation by integrating these signals (Figures 1, 2).

### TRAF6

*Traf6*^−/−^ mice die prematurely within 17–19 days after birth, displaying severe osteopetrosis, splenomegaly, thymic atrophy, and defects in lymph node organogenesis (221, 222). B cells isolated from these mice fail to proliferate in response to anti-CD40 stimulation, indicating that TRAF6 is a mediator of CD40 signaling (222). These *ex vivo* data are consistent with an *in vitro* study showing that a B cell line expressing a CD40 mutant incapable of binding TRAF6 fails to secrete IL-6 and Ig following anti-CD40 stimulation (223). Similarly, in transgenic mice expressing a CD40 mutant incapable of TRAF6 recruitment, the generation of plasma cells, IgG production, and affinity maturation are severely compromised upon antigen challenge (224). B cells from *Traf6*^fl/fl^ *Cd19*^Cre^ mice display defects in proliferation, IL-6 production and phosphorylation of p38 MAP kinase upon stimulation with anti-CD40 Ab (225). *In vivo* antigen challenge also reveals the requirement of TRAF6 in T-dependent production of IgG, generation of long-lived plasma cells, isotype switching, and affinity maturation (225).

In T cells, TRAF6 acts as a negative rheostat. *Traf6*^fl/fl^ *Lck*^Cre^ mice develop a systemic inflammatory disease with increased production of Th2 cytokines, significant expansion of the B cell compartment and elevated serum Ab titers, including anti double-stranded DNA Abs (27). *Traf6*^−/−^ T cells are hyperproliferative and display constitutively active PI3K-AKT signaling. These hyperreactive T cells are refractory to Treg-mediated suppression (27). Additionally, *Traf6*^−/−^ T cells exhibit enhanced Th17 differentiation *in vivo* and *in vitro* in the presence of TGF-β (187). This effect was due to increased responsiveness of *Traf6*^−/−^ T cells to TGF-β as TRAF6 impedes the production of IL-2 (187), which is a known inhibitor of Th17 differentiation (226). TRAF6 can bind to IL-2Rβ and inhibit Jak1 activation induced by IL-2 (34). Modulation of PI3K, TGF-β, and IL-2 signaling pathways by TRAF6 could directly affect T_FH_ differentiation (Figure 1).

## Summary

The differentiation of T_FH_ cells is a complex process controlled by the integration of multiple signals. Many studies support the conclusion that TRAF proteins are important modulators of T-dependent and T-independent humoral responses. TRAFs act through a variety of mechanisms: modulation of TCR signals and integration of costimulatory and cytokine signals. As detailed above, TRAF2, 3, 5, and 6 are the most relevant ones involved in many T_FH_-inducing and T_FH_-antagonistic signaling pathways. However, the exact mechanisms of how each of these TRAF family members contributes to T_FH_ differentiation remain elusive. Precise elucidation of the relevant mechanisms has been challenging for several reasons. First, TRAFs have dual functions as E3 ubiquitin ligases as well as molecular adaptors for protein-protein interactions; second, TRAFs are ubiquitously expressed in innate and adaptive immune cells as well as in non-immune cells; and finally, TRAFs are involved in a variety of signaling pathways that reinforce and/or neutralize each other. All these factors confound the interpretation of results derived from systemic deletion of TRAF proteins *in vivo*.

Future studies addressing TRAF-related mechanisms will be facilitated by the modern genome editing tools that simplify the generation of knock-in or cell type-specific knockout mice. For example, the E3 ligase activity of TRAF proteins could be specifically attenuated using the CRISPR-Cas system to differentiate the enzymatic vs. adaptor function of these proteins in various signaling pathways. Other genomic technologies such as single-cell RNA-Seq and CyTOF could be incorporated into various experiments to simultaneously profile the gene and protein expression alterations of different immune and non-immune cell populations. These approaches will provide a broader perspective of the role of TRAFs in different cell types during an immune response.

Studies on the role of TRAFs in T_FH_ differentiation and B cell responses are of therapeutic interest as modulation of T_FH_ differentiation has the potential to either reduce pathological Ab production in autoimmune and inflammatory diseases, or favor the development of long-lasting and high affinity humoral responses in the context of vaccination or treatment of infectious diseases.

## Author contributions

All authors listed have made a substantial, direct and intellectual contribution to the work, and approved it for publication.

### Conflict of interest statement

The authors declare that the research was conducted in the absence of any commercial or financial relationships that could be construed as a potential conflict of interest.
